# Development and validation of a nomogram for early nutritional risk stratification in elderly patients with pulmonary sepsis: a retrospective cohort study

**DOI:** 10.3389/fnut.2026.1878332

**Published:** 2026-07-31

**Authors:** Xiaoming Yang, Meiyan He, Zhongda Liu, Quan Li, Hui Huang, Lingyong Chen

**Affiliations:** Department of Respiratory and Critical Care Medicine, Lishui Hospital of Traditional Chinese Medicine Affiliated to Zhejiang University of Traditional Chinese Medicine, Lishui, China

**Keywords:** critical care, elderly, LASSO regression, nomogram, NRS-2002, nutritional risk, pulmonary sepsis, risk prediction

## Abstract

**Background:**

Malnutrition is highly prevalent among elderly patients with sepsis and is associated with adverse outcomes; however, early identification of nutritional risk remains challenging. This study aimed to develop and validate a practical prediction model for early nutritional risk in elderly patients with pulmonary sepsis.

**Methods:**

In this retrospective cohort study, 323 elderly patients with pulmonary sepsis were included. Nutritional risk was defined as a Nutritional Risk Screening 2002 (NRS-2002) score of ≥3. Candidate variables collected within 24 h of admission were screened using least absolute shrinkage and selection operator regression, followed by backward stepwise selection. A multivariable logistic regression model was constructed and visualized as a nomogram. Model performance was evaluated using discrimination, calibration, decision curve analysis, comparison with the modified Nutrition Risk in the Critically Ill (mNUTRIC) score, bootstrap internal validation, and sensitivity analysis using proxy Global Leadership Initiative on Malnutrition (GLIM) criteria.

**Results:**

Nutritional risk was identified in 115 patients (35.6%). Seven predictors, age, body mass index, Barthel Index, Sequential Organ Failure Assessment score, serum sodium, albumin, and hemoglobin–albumin–lymphocyte–platelet score, were retained in the final model. The nomogram demonstrated good discrimination (area under the curve [AUC] 0.803, 95% CI 0.755–0.852) and satisfactory calibration (Hosmer–Lemeshow *P* = 0.845). Decision curve analysis indicated a net clinical benefit across a wide range of threshold probabilities (0.15–0.70). Internal validation confirmed model stability (mean AUC, 0.797). The nomogram outperformed the mNUTRIC score in head-to-head comparison (AUC, 0.803 vs. 0.658; *P* < 0.001). Sensitivity analysis using proxy GLIM criteria yielded an AUC of 0.708 (95% CI, 0.650–0.766).

**Conclusion:**

A parsimonious model integrating routinely available clinical variables enables accurate early identification of nutritional risk in elderly patients with pulmonary sepsis. This nomogram may serve as a potential bedside risk-stratification tool, although its use in routine clinical practice requires prospective external validation.

## Introduction

Sepsis, defined as life-threatening organ dysfunction caused by a dysregulated host response to infection, remains a leading cause of mortality in intensive care units (1). With global population aging, both the incidence and mortality of sepsis among older adults continue to rise, posing a substantial public health burden (2). In a multicenter cohort of patients aged ≥65 years, hospital mortality reached 48.8%, highlighting the vulnerability of this population (3).

Malnutrition is highly prevalent among elderly patients with sepsis and is associated with adverse outcomes, including increased mortality, prolonged mechanical ventilation, and higher rates of intensive care admission (4, 5). Its reported incidence ranges from 40% to 60%, depending on the diagnostic criteria (6) used. Mechanistically, sepsis induces a hypermetabolic and catabolic state characterized by systemic inflammation, reduced nutritional intake, and iatrogenic factors such as fasting and ventilatory support, collectively accelerating protein–energy depletion. This process impairs immune function, delays tissue repair, and contributes to a self-reinforcing cycle of clinical deterioration (5). Early identification of individuals at nutritional risk, followed by timely and targeted nutritional support, is therefore critical for improving outcomes in this population.

Despite its clinical importance, nutritional risk in older adults remains under-recognized and frequently underdiagnosed, often being identified only after overt clinical manifestations appear (7). Several tools, including the Nutritional Risk Screening 2002 (NRS-2002), Patient-Generated Subjective Global Assessment, and Global Leadership Initiative on Malnutrition (GLIM) criteria, are widely used in clinical practice (8–10). However, these approaches rely partly on subjective assessment or isolated indicators and may not adequately integrate multiple risk factors into individualized risk prediction (11, 12). Moreover, in elderly patients with pulmonary sepsis, impaired consciousness, delirium, or mechanical ventilation often preclude accurate assessment of recent dietary intake or weight change, limiting the applicability of conventional tools such as NRS-2002. Multivariable prediction models provide an opportunity to overcome these limitations by integrating routinely available clinical variables into quantitative, individualized risk estimates. Least absolute shrinkage and selection operator (LASSO) regression enables efficient variable selection while reducing overfitting, and has been increasingly applied in clinical prediction modeling (13). When translated into a nomogram, such models allow rapid bedside estimation of patient-specific risk, thereby facilitating clinical implementation and decision-making.

To date, no validated model has specifically addressed early nutritional risk in elderly patients with pulmonary sepsis. In this study, we retrospectively analyzed a cohort of such patients and developed a LASSO-based logistic regression model using the NRS-2002 as the reference standard. We further constructed a nomogram based on objective, readily available laboratory and clinical parameters, aiming to provide a practical tool for early risk stratification independent of subjective clinical history.

## Materials and methods

### Ethics

This retrospective cohort study was approved by the Ethics Committee of Lishui Hospital of Traditional Chinese Medicine Affiliated with Zhejiang University of Traditional Chinese Medicine (Approval No. (2023) LSLS-KY-008). The requirement for written informed consent was waived due to the study’s retrospective nature and the use of anonymized data.

### Study population

Consecutive elderly patients with pulmonary sepsis admitted to the Department of Respiratory and Critical Care Medicine, Lishui Hospital of Traditional Chinese Medicine Affiliated with Zhejiang University of Traditional Chinese Medicine, between January 2023 and December 2025, were retrospectively included.

Nutritional risk was assessed using the NRS-2002, with a total score ≥3 indicating nutritional risk. Trained clinicians routinely performed all assessments within 24 h of admission. Patients were stratified into nutritional risk and non-nutritional risk groups. Pulmonary sepsis was defined as sepsis originating from an infectious focus localized to the lungs.

The inclusion criteria were as follows: (1) age ≥60 years; (2) pulmonary sepsis defined according to Sepsis-3 criteria [infection with an acute increase in Sequential Organ Failure Assessment (SOFA) score of ≥2] (1); (3) hospital stay of ≥24 h; and (4) availability of complete clinical data within 24 h of admission. The exclusion criteria were as follows: (1) terminal malignancy or receipt of palliative care; (2) history of gastrointestinal surgery (e.g., gastrectomy or enterectomy); (3) severe hepatic or renal dysfunction (Child–Pugh class C or estimated glomerular filtration rate <30 mL/min/1.73 m^2^); (4) receipt of enteral or parenteral nutrition before admission; (5) pregnancy or lactation; and (6) repeated admissions, for which only the first hospitalization was included.

### Data collection and candidate predictors

Clinical data within 24 h of admission were extracted from the electronic medical record system, including demographic characteristics [age, sex, height, weight, body mass index (BMI), educational level, marital status, level of consciousness, and admission to the respiratory intensive care unit (RICU)], vital signs (temperature, pulse, and respiratory rate), medical history (eight categories, including hypertension, diabetes, cardiovascular disease, cerebrovascular disease, hepatic disease, and renal disease), laboratory parameters (including a complete blood count, metabolic panel, coagulation profile, and inflammatory markers, comprising 60 variables in total); and clinical scores (SOFA score and Barthel Index). All laboratory values corresponded to the first measurement obtained within 24 h after admission. Variable definitions and coding are provided in Supplementary Table 1.

### Sample size estimation

Sample size was evaluated using complementary approaches. Based on the events-per-variable (EPV) principle (≥10 events per predictor), up to 11 predictors could be supported. In addition, an *a priori* power analysis (PASS 15.0), assuming an expected area under the receiver operating characteristic curve (AUC) of 0.75, an event proportion of 0.35, α = 0.05, and β = 0.10, indicated a minimum sample size of 280. The final cohort (*n* = 323) exceeded this requirement (14).

### Statistical analysis

Continuous variables are presented as medians (interquartile ranges), and categorical variables as counts (percentages). Group comparisons were performed using the Wilcoxon rank-sum test or χ^2^ test, as appropriate. Variables with >30% missingness were excluded. The remaining variables had <1% missing data; continuous variables were imputed using the median, and categorical variables were imputed using the mode.

A two-step procedure was applied. First, LASSO regression with 10-fold cross-validation (α = 1) was used to select predictors from the 60 candidate variables. The “lambda.1se” (λ_1se_) criterion was adopted to enhance model parsimony, with convergence limited to 1,000 iterations and a tolerance of 1e^–7^. Variables with nonzero coefficients were retained. Second, these variables were entered into a backward stepwise regression using the Akaike information criterion (AIC) to derive the final set of predictors.

The selected predictors were entered into a multivariable logistic regression model, with nutritional risk as the dependent variable. Odds ratios (ORs) and 95% confidence intervals (CIs) were estimated. Multicollinearity was assessed using the variance inflation factor (VIF), with values >5 indicating multicollinearity.

A nomogram was constructed to visualize the contribution of each predictor. Model discrimination was evaluated using the AUC, and 95% CIs were calculated using the DeLong method. Calibration was assessed using the Hosmer–Lemeshow goodness-of-fit test and calibration curves based on 1,000 bootstrap resamples. Clinical utility was evaluated using decision curve analysis. Internal validation was performed using bootstrap resampling with 1,000 iterations to estimate model optimism, the optimism-corrected AUC, mean AUC, and accuracy. As a complementary internal validation strategy, a split-sample validation was performed by randomly assigning 70% of patients to a training set and 30% to a hold-out test set. Model discrimination and calibration were then evaluated in the test set. Model performance was further evaluated across age subgroups (<70, <80, and <90 years).

For head-to-head comparison, the mNUTRIC score was calculated using available clinical components. Discriminative performance between the nomogram and mNUTRIC score was compared using ROC analysis, and the difference in AUC was tested using DeLong’s test for correlated ROC curves. Clinical utility was compared using decision curve analysis where applicable.

In a sensitivity analysis, model performance was evaluated against a proxy GLIM framework. Because direct measures of muscle mass or muscle function, such as computed tomography-derived skeletal muscle index or handgrip strength, were unavailable, low BMI was used as the phenotypic criterion. Low BMI was defined as BMI < 18.5 kg/m^2^ for patients aged <70 years and <20.0 kg/m^2^ for those aged ≥70 years, consistent with GLIM recommendations for Asian populations. Sepsis was used as the etiologic criterion, reflecting acute inflammation and disease burden, and was present in all enrolled patients. Patients meeting both the low-BMI phenotypic criterion and the etiologic criterion were classified as proxy GLIM-positive.

All analyses were conducted using R software (version 4.5.2). Key packages included *glmnet* for LASSO regression, *rms* for nomogram development, *pROC* for ROC analysis, and *rmda* for decision curve analysis. A two-sided *P* < 0.05 was considered statistically significant.

## Results

### Baseline characteristics

A total of 323 elderly patients with pulmonary sepsis were included, of whom 115 (35.6%) were classified as having nutritional risk and 208 (64.4%) as having no nutritional risk.

Compared with the no nutritional risk group, patients with nutritional risk were older (median, 80 vs. 75 years, *P* < 0.001), had lower BMI (19.72 vs. 20.86 kg/m^2^, *P* < 0.001) and Barthel Index scores (30 vs. 60, *P* < 0.001), and had higher SOFA scores (4 vs. 3, *P* < 0.001). They also exhibited lower serum sodium levels (138.00 vs. 139.75 mmol/L, *P* = 0.023), albumin levels (31.00 vs. 33.00 g/L, *P* < 0.001), and hemoglobin–albumin–lymphocyte–platelet (HALP) scores (10.82 vs. 14.47, *P* = 0.005). No significant differences were observed for the remaining variables (Table 1 and Supplementary Table 2).

**TABLE 1:** Baseline characteristics of elderly patients with pulmonary sepsis according to nutritional risk status.

Variable	Total (*N* = 323)	No nutritional risk (*N* = 208)	Nutritional risk (*N* = 115)	*P-*value
Age, median (IQR), years	78.00 (71.00, 85.00)	75.00 (69.00, 84.00)	80.00 (75.00, 88.00)	<0.001
Gender, *n* (%)				0.838
Male	221 (68%)	141 (68%)	80 (70%)	
Female	102 (32%)	67 (32%)	35 (30%)	
BMI, median (IQR), kg/m^2^	20.31 (19.11, 21.97)	20.86 (19.48, 22.60)	19.72 (18.42, 21.16)	<0.001
Marital status, *n* (%)				0.037
Married	250 (77%)	169 (81%)	81 (70%)	
Other	73 (23%)	39 (19%)	34 (30%)	
Consciousness, *n* (%)				<0.001
Alert	288 (89%)	195 (94%)	93 (81%)	
Impaired	35 (11%)	13 (6.3%)	22 (19%)	
RICU admission, *n* (%)				<0.001
Admitted	89 (28%)	41 (20%)	48 (42%)	
Not admitted	234 (72%)	167 (80%)	67 (58%)	
Barthel index, median (IQR)	50.00 (25.00, 70.00)	60.00 (40.00, 75.00)	30.00 (15.00, 45.00)	<0.001
SOFA score, median (IQR)	4.00 (3.00, 5.00)	3.00 (3.00, 4.00)	4.00 (3.00, 5.00)	<0.001
Malignant tumor, *n* (%)	65 (20%)	34 (16%)	31 (27%)	0.033
Sodium, median (IQR), mmol/L	139.30 (135.60, 142.00)	139.75 (136.05, 142.60)	138.00 (135.10, 141.30)	0.023
Albumin, median (IQR), g/L	32.10 (27.30, 34.60)	33.00 (28.85, 35.05)	31.00 (26.00, 33.70)	<0.001
HALP score, median (IQR)	13.25 (7.66, 23.17)	14.47 (8.22, 26.85)	10.82 (6.51, 18.53)	0.005

Data are presented as median (interquartile range) or *n* (%). *P*-values were calculated using the Wilcoxon rank-sum test for continuous variables and the chi-square test or Fisher’s exact test for categorical variables, as appropriate. BMI, body mass index; HALP, hemoglobin–albumin–lymphocyte–platelet; RICU, respiratory intensive care unit; SOFA, Sequential Organ Failure Assessment.

### Variable selection and model development

Least absolute shrinkage and selection operator regression with 10-fold cross-validation (λ_1se_) identified nine candidate predictors with nonzero coefficients from the initial 60 variables: age, RICU admission, BMI, Barthel Index, SOFA score, malignant tumor, sodium, albumin, and HALP score (Supplementary Figures 1, 2). Subsequent backward stepwise regression based on the Akaike information criterion removed respiratory intensive care unit admission and malignant tumor, yielding a final model comprising seven predictors: age, BMI, Barthel Index, SOFA score, serum sodium, albumin, and HALP score.

### Multivariable analysis

**FIGURE 1:**
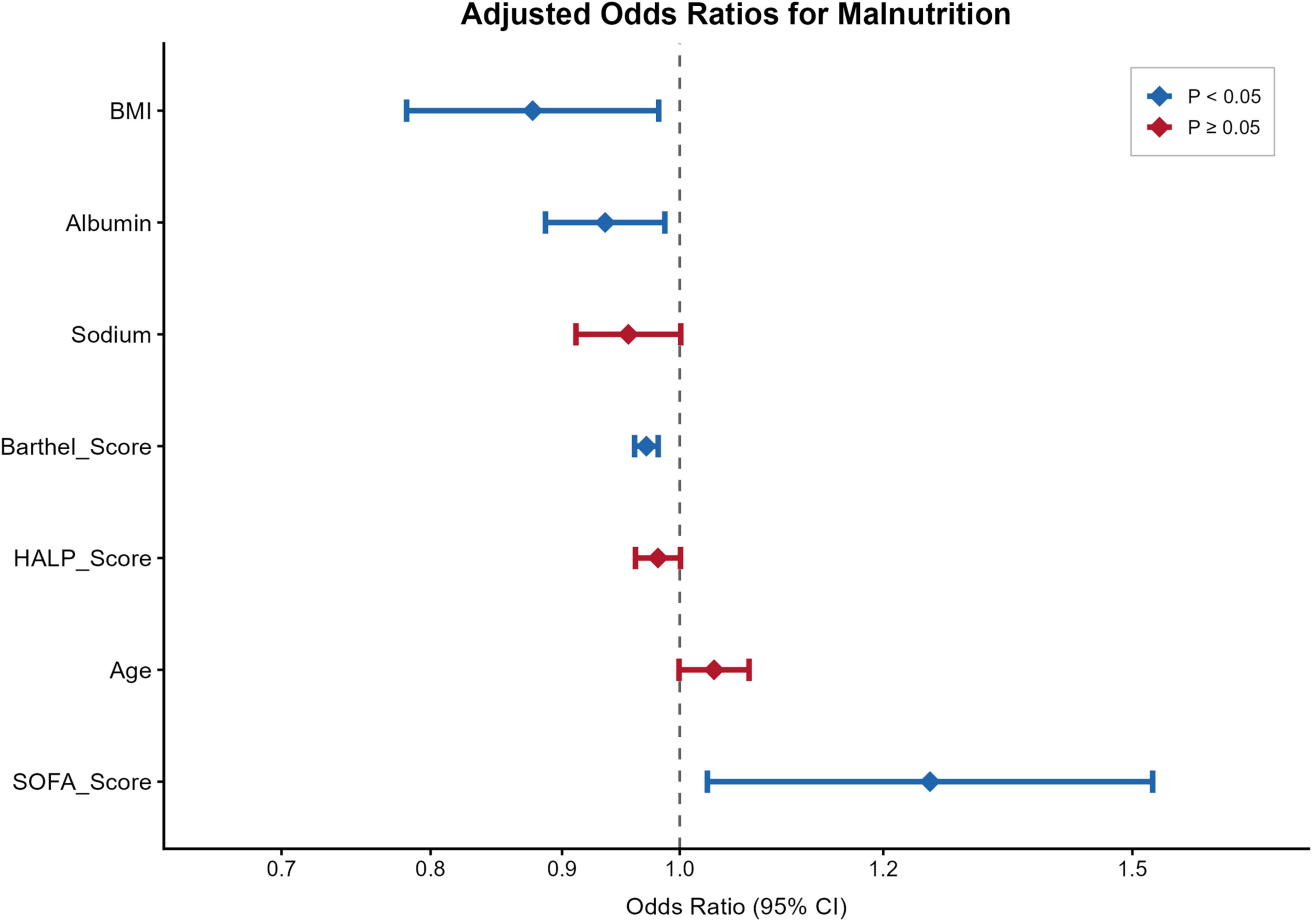
Multivariable logistic regression analysis of factors associated with nutritional risk. Forest plot displaying adjusted odds ratios (ORs) and 95% confidence intervals (CIs) for variables included in the final model. Lower Barthel Index, lower body mass index (BMI), and lower albumin levels were independently associated with increased odds of nutritional risk, whereas higher Sequential Organ Failure Assessment (SOFA) scores were associated with elevated risk. Age, serum sodium, and hemoglobin–albumin–lymphocyte–platelet (HALP) score showed borderline associations with the outcome. The vertical dashed line indicates an OR of 1.0.

The results of the multivariable logistic regression analysis are presented in Figure 1. In the multivariable logistic regression model, lower Barthel Index (OR = 0.97, 95% CI: 0.96–0.98, *P* < 0.001), lower albumin (OR = 0.94, 95% CI: 0.89–0.99, *P* = 0.014), and lower BMI (OR = 0.88, 95% CI: 0.78–0.98, *P* = 0.022) were independently associated with an increased risk of nutritional risk, whereas a higher SOFA score was associated with an increased risk (OR = 1.25, 95% CI: 1.03–1.53, *P* = 0.028). Age (OR = 1.03, *P* = 0.054), sodium (OR = 0.96, *P* = 0.055), and HALP score (OR = 0.98, *P* = 0.058) showed borderline associations with the outcome. No evidence of multicollinearity was observed (all VIFs < 1.1; Supplementary Table 3). The full results of the multivariable analysis are presented in Table 2.

**TABLE 2:** Multivariable logistic regression analysis of factors associated with nutritional risk.

Variable	β	SE	OR	95% CI	*P*-value
Age, years	0.031	0.016	1.03	1.00–1.06	0.054
BMI, kg/m^2^	−0.132	0.058	0.88	0.78–0.98	0.022
Barthel Index	−0.030	0.005	0.97	0.96–0.98	<0.001
SOFA score	0.224	0.102	1.25	1.03–1.53	0.028
Sodium, mmol/L	−0.046	0.024	0.96	0.91–1.00	0.055
Albumin, g/L	−0.067	0.027	0.94	0.89–0.99	0.014
HALP score	−0.019	0.010	0.98	0.96–1.00	0.058

Odds ratios and 95% confidence intervals were estimated using multivariable logistic regression. A two-sided *P* < 0.05 was considered statistically significant. β, regression coefficient; CI, confidence interval; OR, odds ratio; SE, standard error; BMI, body mass index; SOFA, Sequential Organ Failure Assessment; HALP, hemoglobin–albumin–lymphocyte–platelet.

### Model performance and validation

**FIGURE 2:**
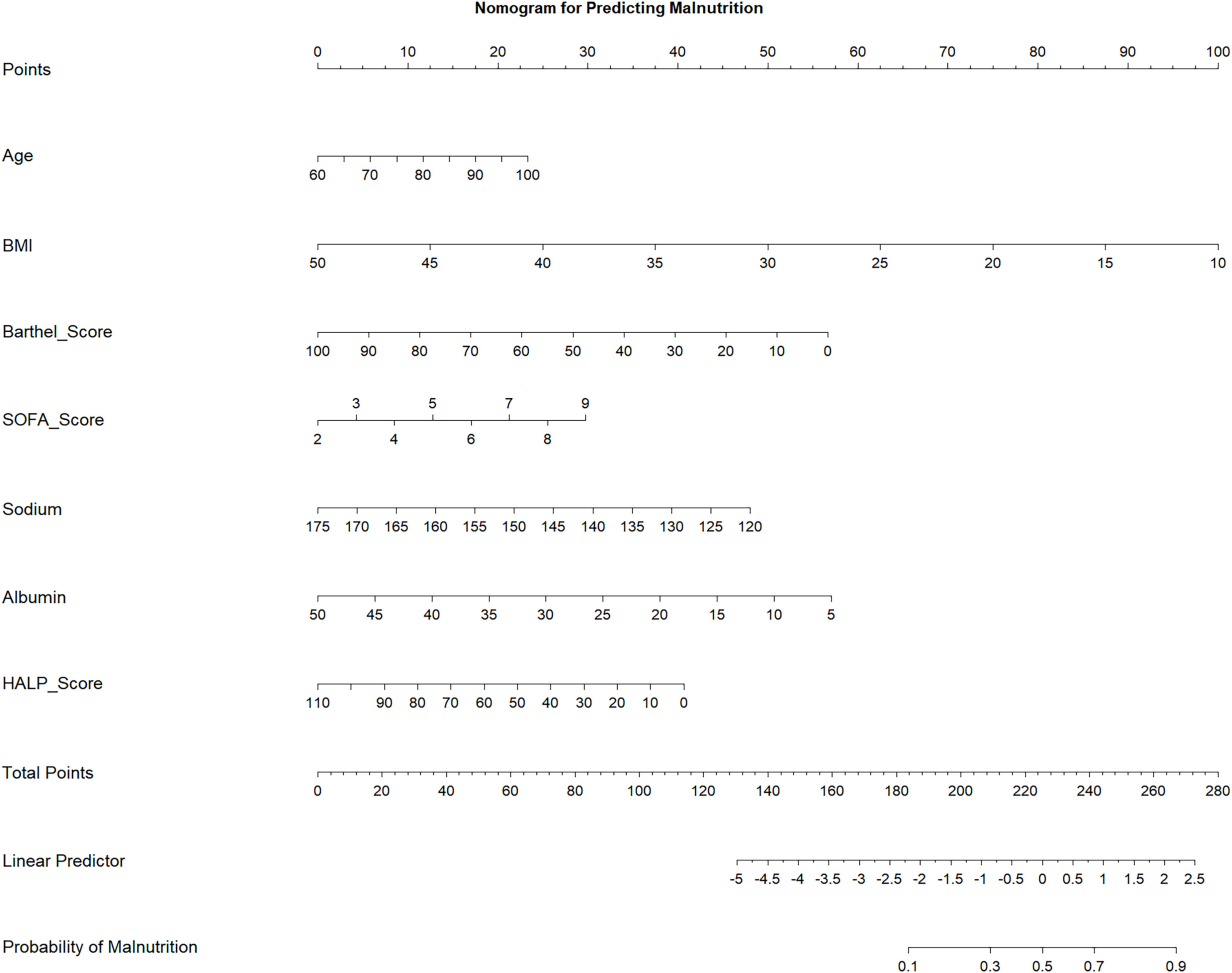
Nomogram for individualized prediction of nutritional risk. Nomogram derived from the multivariable logistic regression model to estimate the probability of nutritional risk in elderly patients with pulmonary sepsis. Each predictor is assigned a weighted score proportional to its regression coefficient. The sum of individual scores corresponds to the total points, which map to the predicted probability of nutritional risk on the lower scale. This tool facilitates individualized risk estimation at the bedside.

**FIGURE 3:**
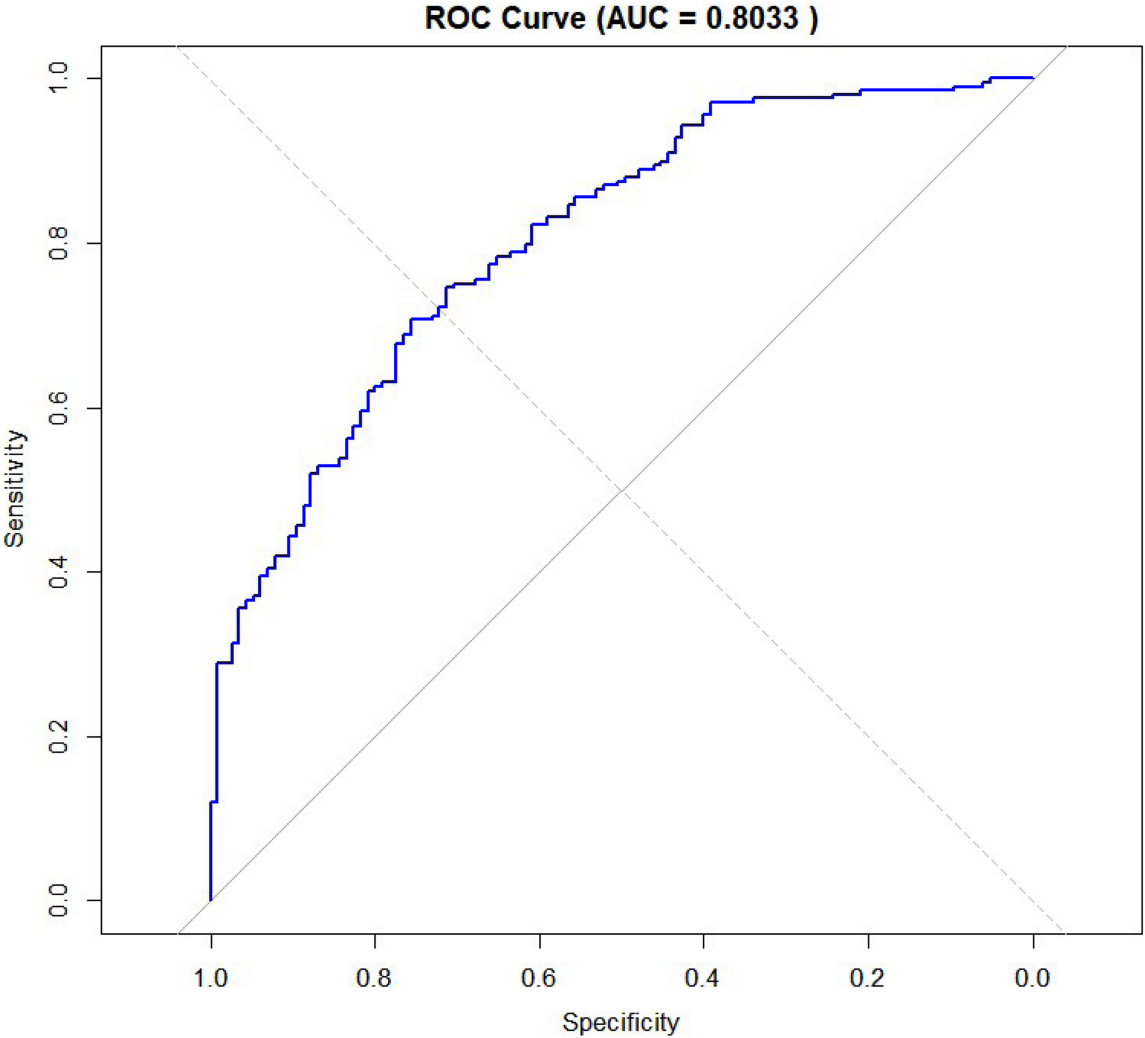
Discriminative performance of the prediction model. Receiver operating characteristic (ROC) curve illustrating the discriminative ability of the multivariable model for predicting nutritional risk. The area under the curve (AUC) was 0.803 (95% confidence interval [CI] 0.755–0.852), indicating good discrimination.

**FIGURE 4:**
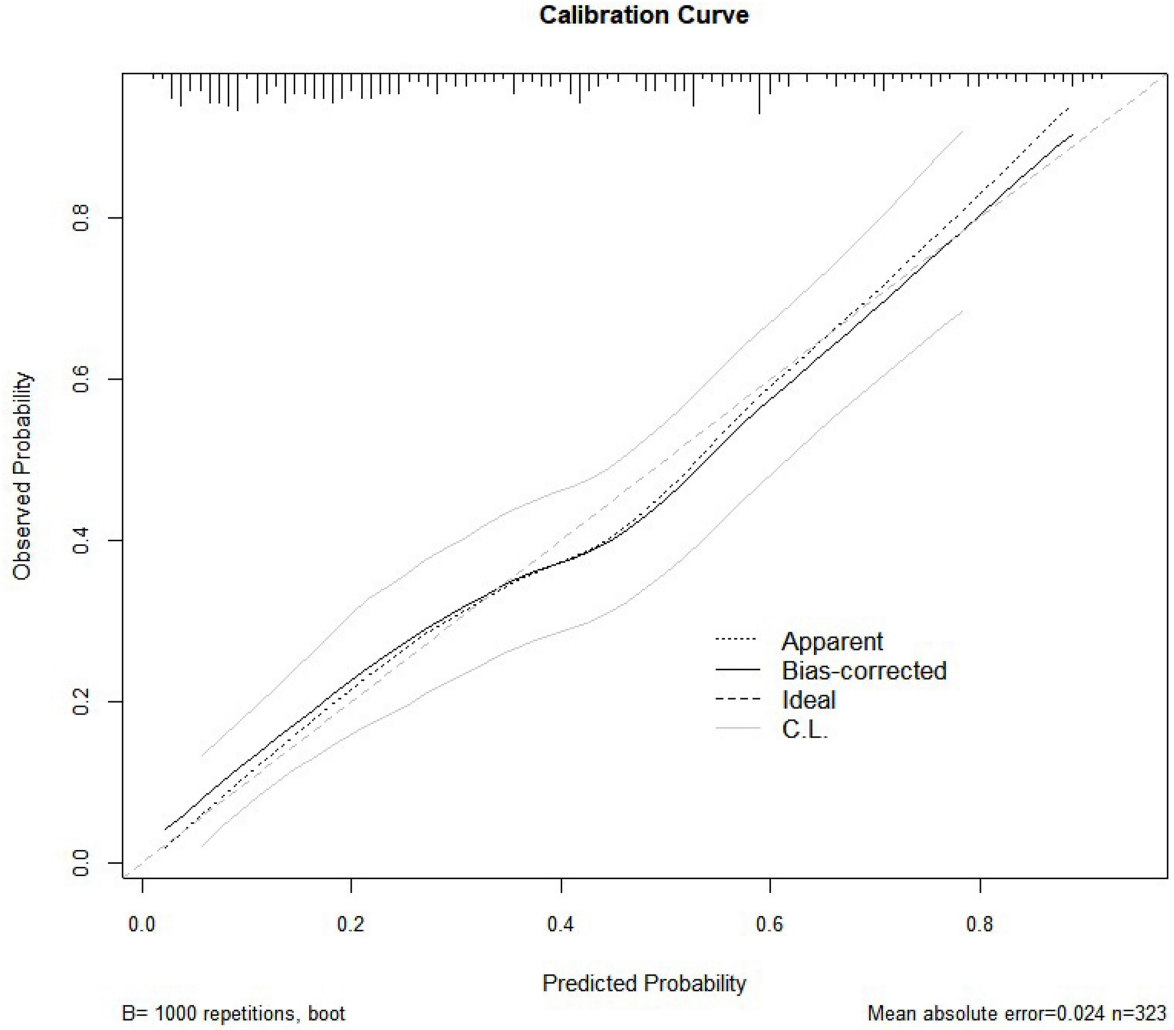
Calibration of the prediction model. Calibration curve comparing predicted and observed probabilities of nutritional risk. The bias-corrected curve shows good agreement with the ideal reference line, indicating adequate calibration. The Hosmer–Lemeshow test was non-significant (*P* = 0.845).

A nomogram was constructed based on the final multivariable model (Figure 2). The model demonstrated good discrimination, with an AUC of 0.803 (95% CI: 0.755–0.852) (Figure 3). Calibration was satisfactory, as indicated by a non-significant Hosmer–Lemeshow test (*P* = 0.845) and good agreement between predicted and observed probabilities (Figure 4).

**FIGURE 5:**
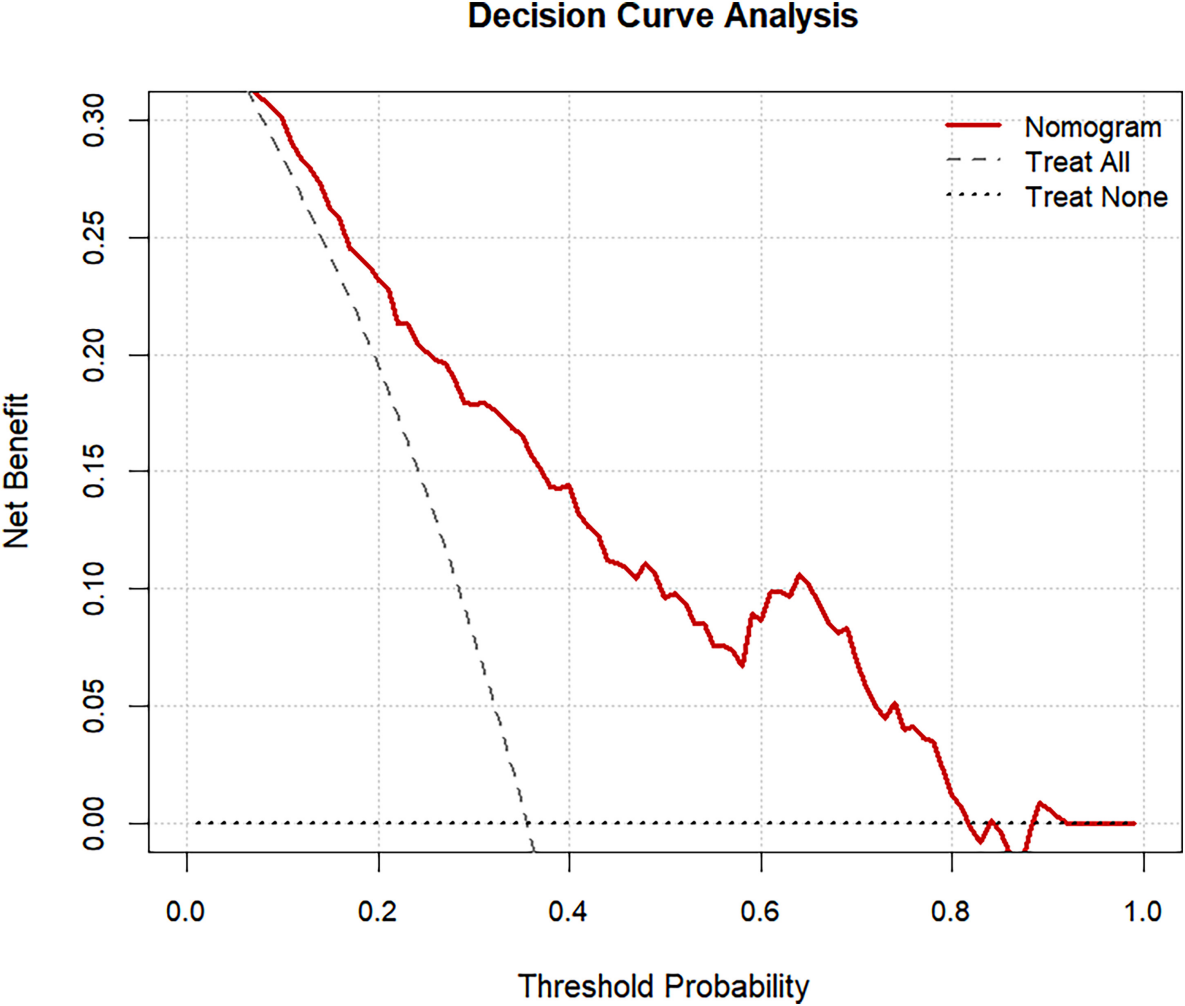
Clinical utility of the prediction model assessed by decision curve analysis. Decision curve analysis (DCA) showing the net clinical benefit of the multivariable model for predicting nutritional risk across a range of threshold probabilities. The model demonstrates a higher net benefit than the “treat-all” and “treat-none” strategies within a threshold probability range of approximately 0.15–0.70.

Decision curve analysis showed that the model provided a net clinical benefit across a threshold probability range of 0.15–0.70 compared with treat-all and treat-none strategies (Figure 5). Bootstrap internal validation with 1,000 resamples yielded a mean AUC of 0.797 (95% CI: 0.785–0.804) and an optimism-corrected AUC of 0.785, with estimated optimism of 0.018. The mean accuracy was 0.712 (95% CI, 0.681–0.740). The similarity between the apparent AUC, bootstrap mean AUC, and optimism-corrected AUC suggested limited overfitting and stable internal performance (Supplementary Table 4). In the complementary split-sample validation, the model achieved an AUC of 0.779 (95% CI, 0.683–0.875) in the hold-out test set (*n* = 96), with acceptable calibration (Supplementary Figures 7, 8 and Supplementary Table 9).

In sensitivity analysis using proxy GLIM criteria, defined by low BMI as the phenotypic criterion and sepsis as the etiologic criterion, the nomogram yielded an AUC of 0.708 (95% CI, 0.650–0.766; Supplementary Figure 3 and Supplementary Table 5). This finding suggests moderate agreement between the model and a BMI-based proxy definition of malnutrition.

### Comparative and subgroup analyses

The combined model demonstrated superior discriminative performance (AUC = 0.803) compared with the individual predictors (AUC range: 0.576–0.752), with the Barthel Index showing the highest performance among the single predictors (AUC = 0.752) (Supplementary Figure 4).

In age-stratified analyses, the model maintained consistent performance across all subgroups, with AUC values exceeding 0.75. The highest discrimination was observed in patients aged <80 years (AUC = 0.834), followed by those aged <70 years (AUC = 0.833) and those aged <90 years (AUC = 0.806), indicating robust performance across different age strata (Supplementary Figure 5 and Supplementary Table 6).

To further assess model robustness, age-stratified multivariable analyses (<80 vs. ≥80 years) were performed using the same predictors (Supplementary Figure 6). The direction and magnitude of associations for most variables—including BMI, Barthel Index, SOFA score, albumin, sodium, and HALP score—were broadly consistent across age groups. A significant interaction between age and age group was identified (Type II Wald χ^2^, *P* = 0.011). Age was independently associated with nutritional risk in patients aged <80 years (OR per year = 1.13, 95% CI 1.03–1.24, *P* = 0.010), but not in those aged ≥80 years (OR = 0.995, 95% CI 0.92–1.08, *P* = 0.906), suggesting attenuation of its predictive value in the oldest subgroup. No significant interactions were observed for other predictors (all *P* > 0.05).

In direct comparison, the nomogram demonstrated better discrimination than the mNUTRIC score (AUC, 0.803 vs. 0.658; DeLong test, *P* < 0.001; Supplementary Table 7). Decision curve analysis also indicated greater net clinical benefit of the nomogram than the mNUTRIC score across clinically relevant threshold probabilities.

## Discussion

Malnutrition remains highly prevalent among older adults, particularly those requiring hospitalization. It is consistently associated with worse clinical outcomes, including increased mortality and prolonged recovery (15, 16). However, only a minority of malnourished older adults are diagnosed and receive nutritional support (17). These gaps highlight the need for practical, objective tools that enable early identification of high-risk individuals in routine clinical settings. In this study, a multivariable nomogram was developed and internally validated to predict early nutritional risk in elderly patients with pulmonary sepsis. Among 323 patients, 115 (35.6%) were classified as being at nutritional risk. From 60 candidate variables, seven predictors, age, BMI, Barthel Index, SOFA score, serum sodium, albumin, and HALP score, were retained. The model demonstrated good discrimination (AUC 0.803, 95% CI 0.755–0.852) and calibration (Hosmer–Lemeshow *P* = 0.845), with consistent performance on bootstrap validation (mean AUC 0.797). Decision curve analysis further indicated a clinically meaningful net benefit across threshold probabilities of 0.15–0.70. Collectively, these findings suggest that integrating routinely available clinical variables can yield clinically useful risk stratification. The head-to-head comparison with mNUTRIC further suggests that the proposed nomogram may offer incremental discriminative value for identifying NRS-2002-defined nutritional risk in elderly patients with pulmonary sepsis. However, because NRS-2002 was used as the outcome definition, this comparison should be interpreted as prediction of screening-defined nutritional risk rather than diagnosis of malnutrition.

Age showed a borderline association with nutritional risk (OR 1.03, *P* = 0.054), but its effect was not uniform across subgroups. A significant interaction with age stratification (*P*_*interaction*_ = 0.011) indicated that age was predictive in patients aged <80 years but not in those aged ≥80 years, suggesting attenuation of its prognostic value in the oldest subgroup. This may reflect reduced variability and competing risks in very elderly individuals, where frailty and disease burden predominate. Notably, model performance remained stable across age strata (AUC > 0.75), with optimal discrimination observed in patients aged <80 years (AUC 0.834). Although respiratory intensive care unit admission and malignant tumor were associated with nutritional risk in univariable analyses, they were not retained in the final multivariable model. This likely reflects attenuation of their independent predictive contribution after accounting for stronger clinical and biochemical predictors, such as SOFA score, functional status, albumin, and HALP score, rather than a primary effect of statistical collinearity. The combined LASSO and backward-selection procedure favored a parsimonious predictor set while preserving overall model performance. These findings align with evidence that aging-related immune dysregulation and reduced physiological reserve contribute to both sepsis severity and nutritional vulnerability (18, 19). However, they also highlight that chronological age alone is insufficient to capture risk heterogeneity in very elderly populations, necessitating multidimensional assessment.

Body mass index was independently associated with nutritional risk (OR 0.88, 95% CI 0.78–0.98), reflecting reduced energy reserves in underweight individuals. In a large cohort of 6,602 patients with sepsis, a U-shaped relationship between BMI and 28-day mortality was observed, with particularly adverse outcomes among elderly patients with low BMI (20). However, BMI has well-recognized limitations in critical illness. Fluid resuscitation and capillary leak can lead to edema, artificially inflating body weight and overestimating lean body mass (21, 22). Moreover, BMI does not capture sarcopenia, which is highly prevalent in sepsis and strongly associated with poor outcomes (23). These limitations support the inclusion of complementary functional and biochemical markers in predictive models.

The Barthel Index emerged as the strongest protective factor (OR 0.97, 95% CI 0.96–0.98), highlighting the importance of functional status. Impairment in activities of daily living reduces the ability to maintain adequate nutritional intake and increases vulnerability to catabolic stress. Previous studies have similarly identified functional impairment as an independent predictor of nutritional risk and adverse outcomes in older adults (24, 25). In sepsis populations, functional status has also been shown to have prognostic value. For example, the Barthel Index at ICU discharge independently predicted long-term functional impairment or mortality (OR 0.96, 95% CI 0.95–0.98) (26). Additionally, hospital-acquired functional decline occurs in up to 42.5% of patients with sepsis and is strongly associated with lower baseline mobility (27). These findings reinforce the critical importance of integrating functional assessment into nutritional risk prediction, particularly in elderly populations where functional decline often precedes and exacerbates nutritional deterioration.

The SOFA score was positively associated with nutritional risk (OR 1.25, 95% CI 1.03–1.53), reflecting the interplay between organ dysfunction and metabolic stress. Sepsis induces a hypermetabolic, catabolic state characterized by increased protein breakdown and impaired nutrient utilization (28). Concurrent gastrointestinal dysfunction may further limit nutrient intake, creating a self-perpetuating cycle of nutritional risk and organ failure. Higher SOFA scores have consistently been linked to mortality risk (29), and correlate with established nutritional risk tools such as the modified Nutrition Risk in the Critically Ill score (30). These observations support the concept that nutritional vulnerability and organ dysfunction are inextricably linked in sepsis, and that patients with higher SOFA scores warrant particularly vigilant nutritional surveillance and early intervention. Recent evidence further supports the prognostic relevance of nutritional risk assessment in critically ill patients. For example, Duran and Kilinç reported that the mNUTRIC score predicted survival outcomes in ICU patients, suggesting that combining illness-severity measures with nutritional parameters may improve early risk stratification (31).

Serum sodium showed a borderline association (OR 0.96, *P* = 0.055). Hyponatremia is common in sepsis and reflects complex pathophysiological processes, including inappropriate antidiuretic hormone secretion and renal dysfunction (32, 33). Beyond its neurological manifestations (34), hyponatremia is often accompanied by gastrointestinal symptoms such as nausea and vomiting, which may impair oral intake and exacerbate nutritional deficits. Although not independently significant, its inclusion in the model likely captures additional dimensions of systemic illness severity not fully reflected by other covariates.

Albumin was independently associated with nutritional risk (OR 0.94, 95% CI 0.89–0.99). In sepsis, hypoalbuminemia reflects both nutritional depletion and inflammation-driven redistribution, as albumin functions as a negative acute-phase reactant (5, 35). A meta-analysis of 90 cohort studies (*n* = 291,433) reported that each 10 g/L decrease in albumin was associated with a 137% increase in mortality and a 71% increase in length of stay (36). Beyond its role as a biomarker, albumin contributes to endothelial integrity, antioxidant defense, and molecular transport (37). In sepsis, reduced albumin levels are closely linked to endothelial dysfunction and adverse outcomes, including mortality and bleeding risk (38).

The HALP score, a composite index integrating hemoglobin, albumin, lymphocytes, and platelets, showed a borderline protective association (OR 0.98, *P* = 0.058). This multidimensional marker reflects the intersection of nutrition, inflammation, and immune status. In a large retrospective study of 16,625 patients with sepsis, a nonlinear association between HALP and mortality was observed, with each unit increase below a threshold of 24.69 associated with a 2% reduction in 28-day mortality (HR 0.98, 95% CI 0.97–0.99) (39). Although HALP shares components with albumin and lymphocyte count, variance inflation factors in the present multivariable model were all below 1.1, indicating no evidence of statistical multicollinearity (Supplementary Table 3). HALP may therefore provide additional, although borderline, predictive information beyond albumin alone by integrating hemoglobin, platelets, and immune-cell parameters into a broader nutrition–inflammation–immunity profile. However, because HALP includes albumin as one of its components, conceptual overlap with albumin cannot be fully excluded despite the absence of statistical multicollinearity. This finding should therefore be interpreted cautiously and requires confirmation in external cohorts. To further address potential confounding by systemic inflammation, a sensitivity analysis was performed with additional adjustment for log-transformed high-sensitivity C-reactive protein and procalcitonin. The effect estimates and significance levels of all seven predictors remained materially unchanged (Supplementary Table 8), and model discrimination was nearly identical after adjustment (AUC, 0.8045 vs. 0.8033 in the original model). These findings suggest that the model’s predictive signal was not fully explained by measured acute inflammatory markers.

From a clinical perspective, the model demonstrated good discrimination and calibration, with a clear net benefit on decision curve analysis. Importantly, recent updates in sepsis management guidelines have emphasized individualized, risk-based decision-making rather than rigid protocol-driven approaches (40). In this context, the present nomogram may serve as a practical adjunct for early risk stratification, enabling targeted nutritional and supportive interventions. Compared with previously published sepsis-related nutritional prediction models, the present nomogram differs in its target population, outcome definition, and predictor structure. Whereas prior models have often focused on mortality prediction or broader critically ill populations, our model was developed specifically for early identification of NRS-2002-defined nutritional risk in elderly patients with pulmonary sepsis. The inclusion of the Barthel Index adds a functional-status dimension that is particularly relevant in older adults, in whom impaired activities of daily living may precede or exacerbate nutritional deterioration. By integrating functional assessment with routinely available clinical and biochemical variables, the model provides a pragmatic bedside framework for early nutritional risk stratification when subjective dietary history or recent weight-change information is unavailable or unreliable.

Several limitations should be acknowledged. First, this was a single-center retrospective study. Although bootstrap validation and split-sample validation suggested acceptable internal stability, both approaches were based on the same source population and therefore cannot establish model transportability. The retrospective design may have introduced selection bias, information bias from routinely collected medical records, and residual confounding from unmeasured variables. In addition, because all patients were enrolled from a single institution, the model may reflect local case mix, nutritional screening practice, treatment patterns, and laboratory testing protocols. Accordingly, the present nomogram should be regarded as hypothesis-generating rather than ready for broad clinical implementation. Prospective multicenter validation in geographically and clinically distinct cohorts is required before routine use. Second, the model was based on variables measured within 24 h of admission and did not capture the dynamic evolution of nutritional risk, organ dysfunction, inflammatory status, electrolyte disturbance, or nutritional biomarkers during hospitalization. Serial assessments during the first 72–96 h may improve risk reclassification and better reflect the evolving metabolic response to sepsis, but such dynamic laboratory and clinical data were unavailable in this retrospective dataset. Third, several determinants of nutritional status, including detailed dietary intake, social support, psychological status, pre-admission weight trajectory, prior malnutrition history, chronic inflammatory conditions, and longitudinal weight loss, were not captured. These omissions may have contributed to residual confounding and limited our ability to distinguish acute illness-related nutritional risk from pre-existing nutritional vulnerability. Fourth, the primary outcome was NRS-2002-defined nutritional risk rather than full GLIM-defined malnutrition. Although this outcome was selected to support early risk stratification in patients for whom subjective dietary history may be unreliable or unavailable, it remains a screening-defined endpoint and should not be interpreted as a diagnostic definition of malnutrition. Fifth, although a sensitivity analysis was performed using proxy GLIM criteria, definitive GLIM-based malnutrition diagnosis requires direct assessment of muscle mass or muscle function, such as computed tomography-derived skeletal muscle index or handgrip strength, which was not available. In addition, because BMI was included both as a predictor in the nomogram and as the phenotypic criterion in the proxy GLIM definition, this sensitivity analysis should be interpreted as exploratory and may overestimate the apparent agreement with GLIM-related malnutrition risk. Sixth, components of the HALP score may be influenced by acute inflammatory responses. Although additional adjustment for high-sensitivity C-reactive protein and procalcitonin did not materially change model performance, these markers cannot fully account for chronic inflammatory burden or other unmeasured biological confounders. The borderline association of HALP should therefore be interpreted cautiously and requires confirmation in external cohorts.

Future research should prioritize prospective multicenter validation using independent cohorts with harmonized nutritional assessment procedures. Development of models based on complete GLIM-defined malnutrition, including direct measures of muscle mass or muscle function, is warranted to enhance diagnostic relevance. In addition, incorporation of serial laboratory measurements, functional trajectories, inflammatory biomarkers, and metabolomic profiles may improve risk reclassification. Interventional studies are ultimately needed to determine whether model-guided nutritional strategies improve patient-centered outcomes, including mortality, hospital stay, and functional recovery. Finally, integration of the model into user-friendly digital platforms, such as web-based calculators or mobile applications, may facilitate real-time clinical implementation.

## Conclusion

In this retrospective cohort of elderly patients with pulmonary sepsis, we developed and internally validated a parsimonious seven-variable nomogram for early identification of nutritional risk based on the NRS-2002. The model demonstrated good discrimination, satisfactory calibration, and net clinical benefit, supporting its potential role as a pragmatic bedside tool for risk stratification when comprehensive nutritional assessment is impractical. Key contributors—including the Barthel Index, SOFA score, and albumin—highlight the multifactorial nature of nutritional vulnerability in this population, encompassing functional status, organ dysfunction, and biochemical markers. Despite these strengths, the findings should be interpreted with caution given the single-center design, lack of external validation, and reliance on a screening-based outcome rather than a diagnostic framework for malnutrition. Future studies should prioritize multicenter prospective validation and evaluate models based on GLIM-defined malnutrition to enhance clinical relevance. Ultimately, whether model-guided nutritional strategies translate into improved patient-centered outcomes warrants confirmation in interventional studies.

## Data Availability

The raw data supporting the conclusions of this article will be made available by the authors, without undue reservation.
