# Supplementary material for: Development and validation of a nomogram for early nutritional risk stratification in elderly patients with pulmonary sepsis: a retrospective cohort study
Source: Front Nutr. 2026 Jul 31;13:1878332. doi: 10.3389/fnut.2026.1878332 (PMC13472832; doi:10.3389/fnut.2026.1878332)
Supplement: Supplementary file 1 [file Data_Sheet_1.pdf]

Supplementary Table S1. Definitions and coding of candidate variables used for model development.

Variable	Description	Type	Assignment / Unit
**Outcome variable**			
Malnutrition	Malnutrition status (NRS-2002 ≥ 3)	Dichotomous	0 = No, 1 = Yes
**Demographic characteristics**			
Age	Age	Continuous	Years
Gender	Gender	Dichotomous	Male, Female
**Vital signs**			
Temperature	Body temperature	Continuous	°C
Pulse	Pulse rate	Continuous	beats/min
Respiration	Respiratory rate	Continuous	breaths/min
**Medical history**			
Heart_Disease	History of heart disease	Dichotomous	Yes, No
Hypertension	History of hypertension	Dichotomous	Yes, No
Diabetes	History of diabetes	Dichotomous	Yes, No
Cerebrovascular_Disease	History of cerebrovascular disease	Dichotomous	Yes, No
Liver_Disease	History of liver disease	Dichotomous	Yes, No
Kidney_Disease	History of kidney disease	Dichotomous	Yes, No
Malignant_Tumor	History of malignant tumor	Dichotomous	Yes, No
Osteoporosis	History of osteoporosis	Dichotomous	Yes, No
**Anthropometric measurements**			
Height	Height	Continuous	m
Weight	Body weight	Continuous	kg
BMI	Body mass index	Continuous	kg/m^2
**Clinical scores**			
Barthel_Score	Barthel Index (activities of daily living)	Continuous	0–100
SOFA_Score	Sequential Organ Failure Assessment score	Continuous	0–24
**Laboratory parameters**			

Variable	Description	Type	Assignment / Unit
BNP	B-type natriuretic peptide	Continuous	pg/mL
Calcium	Serum calcium	Continuous	mmol/L
Sodium	Serum sodium	Continuous	mmol/L
Chloride	Serum chloride	Continuous	mmol/L
Potassium	Serum potassium	Continuous	mmol/L
Creatinine	Serum creatinine	Continuous	μmol/L
Procalcitonin	Procalcitonin	Continuous	ng/mL
D_Dimer	D-dimer	Continuous	mg/L
Uric_Acid	Uric acid	Continuous	μmol/L
Phosphorus	Serum phosphorus	Continuous	mmol/L
Magnesium	Serum magnesium	Continuous	mmol/L
Total_Protein	Total protein	Continuous	g/L
Albumin	Albumin	Continuous	g/L
Globulin	Globulin	Continuous	g/L
Prealbumin	Prealbumin	Continuous	mg/L
ALT	Alanine aminotransferase	Continuous	U/L
AST	Aspartate aminotransferase	Continuous	U/L
LDH	Lactate dehydrogenase	Continuous	U/L
CK	Creatine kinase	Continuous	U/L
Glucose	Blood glucose	Continuous	mmol/L
Hemoglobin	Hemoglobin	Continuous	g/L
Hs_CRP	High-sensitivity C-reactive protein	Continuous	mg/L
Neutrophil_Lymphocyte_Ratio	Neutrophil-to-lymphocyte ratio	Continuous	Ratio
Platelet_Count	Platelet count	Continuous	$\times 10^9$/L
WBC_Count	White blood cell count	Continuous	$\times 10^9$/L
pH_Corrected	Temperature-corrected pH	Continuous	-
PaO_2	Temperature-corrected partial pressure of oxygen	Continuous	mmHg
$PaCO_2$	Temperature-corrected partial pressure of carbon dioxide	Continuous	mmHg

Variable	Description	Type	Assignment / Unit
Lactate	Lactate concentration	Continuous	mmol/L
SpO_2	Oxygen saturation	Continuous	%
Triglycerides	Triglycerides	Continuous	mmol/L
Total_Cholesterol	Total cholesterol	Continuous	mmol/L
LDL_Cholesterol	Low-density lipoprotein cholesterol	Continuous	mmol/L
HDL_Cholesterol	High-density lipoprotein cholesterol	Continuous	mmol/L
RDW_Albumin_Ratio	Red blood cell distribution width-to-albumin ratio	Continuous	Ratio
HALP_Score	HALP score[i]	Continuous	Score
Monocyte_Albumin_Ratio	Monocyte-to-albumin ratio	Continuous	Ratio
Neutrophil_Albumin_Ratio	Neutrophil-to-albumin ratio	Continuous	Ratio

[i]HALP_Score calculation: HALP score = (Hemoglobin [g/L] × Albumin [g/L] × Lymphocyte count [$\times 10^9$/L]) / Platelet count [$\times 10^9$/L]. All parameters were obtained from the first blood test within 24 hours of admission.

Note: All laboratory parameters were collected from the first blood test within 24 hours of admission. Continuous variables were analyzed using actual measured values with units as indicated. Categorical variables were analyzed as factors with the levels shown.

Supplementary Table S2. Baseline characteristics not included in the final model.

Variable	No nutritional risk (n = 208)	Nutritional risk (n = 115)	P value
Demographics			
Height, cm	164.0 (158.0–168.0)	165.0 (157.0–168.0)	0.708
Education level, n (%)			0.996
Primary school or below	164 (78.8)	91 (79.1)	
Junior high school	28 (13.5)	16 (13.9)	
Senior high school	12 (5.8)	6 (5.2)	
University or above	4 (1.9)	2 (1.7)	
Vital signs			
Temperature, °C	37.00 (36.65–37.20)	36.90 (36.50–37.40)	0.537
Pulse, beats/min	96.5 (83.5–108.0)	96.0 (79.0–114.0)	0.693
Respiratory rate, breaths/min	21.0 (20.0–22.0)	20.0 (20.0–22.0)	0.329
SpO_2, %	97.5 (95.5–98.9)	96.9 (95.0–98.7)	0.320
Comorbidities, n (%)			
Heart disease	71 (34.1)	39 (33.9)	>0.999
Hypertension	62 (29.8)	36 (31.3)	0.878
Osteoporosis	22 (10.6)	18 (15.7)	0.250
Diabetes mellitus	21 (10.1)	17 (14.8)	0.284
Cerebrovascular disease	47 (22.6)	26 (22.6)	>0.999
Liver disease	39 (18.8)	23 (20.0)	0.900
Kidney disease	48 (23.1)	26 (22.6)	>0.999
Electrolytes			
Potassium, mmol/L	4.08 (3.64–4.50)	4.07 (3.63–4.67)	0.703
Chloride, mmol/L	102.20 (98.15–106.05)	101.20 (95.70–104.80)	0.064
Calcium, mmol/L	2.15 (2.06–2.26)	2.16 (2.07–2.28)	0.772
Phosphorus, mmol/L	0.97 (0.80–1.14)	1.02 (0.83–1.19)	0.251
Magnesium, mmol/L	0.84 (0.77–0.92)	0.81 (0.75–0.92)	0.119
Hepatic and renal function			
Creatinine, µmol/L	80.0 (59.6–110.0)	78.0 (51.8–112.6)	0.390
Uric acid, µmol/L	239.0 (182.0–333.0)	223.0 (154.0–372.0)	0.306
ALT, U/L	20.0 (13.0–39.0)	24.0 (13.0–41.0)	0.545
AST, U/L	26.0 (19.0–42.0)	30.0 (20.0–54.0)	0.107
LDH, U/L	247.0 (198.0–338.5)	232.0 (189.0–397.0)	0.912
CK, U/L	60.5 (35.0–132.5)	55.0 (32.0–87.0)	0.100
Nutritional and protein markers			
Total protein, g/L	61.25 (56.00–65.90)	60.50 (55.00–65.50)	0.416
Globulin, g/L	29.85 (25.20–33.80)	29.50 (25.60–34.00)	0.713
Prealbumin, mg/L	120.50 (83.85–173.59)	119.07 (77.00–151.07)	0.303
Inflammatory and hematologic markers			
WBC count, $\times 10^9$/L	8.65 (6.00–12.45)	9.20 (7.21–12.99)	0.227
Platelet count, $\times 10^9$/L	186.5 (134.0–280.5)	203.0 (131.0–271.0)	0.727
Procalcitonin, ng/mL	0.80 (0.20–4.01)	0.98 (0.30–3.59)	0.307
hs-CRP, mg/L	60.66 (21.55–128.72)	72.90 (25.80–130.17)	0.387

Variable	No nutritional risk (n = 208)	Nutritional risk (n = 115)	P value
D-dimer, mg/L	1205.0 (428.5–2895.0)	1460.0 (434.0–3680.0)	0.282
BNP, pg/mL	204.0 (76.1–429.3)	191.0 (94.0–448.9)	0.643
Glucose, mmol/L	7.10 (5.76–9.10)	7.53 (5.77–10.40)	0.287
Arterial blood gas analysis			
pH	7.42 (7.38–7.45)	7.42 (7.39–7.45)	0.396
PaO_2, mmHg	84.1 (71.5–109.6)	81.1 (69.9–101.5)	0.412
$PaCO_2$, mmHg	37.2 (32.7–44.8)	36.8 (32.7–43.0)	0.427
Lactate, mmol/L	1.70 (1.30–2.20)	1.50 (1.20–2.10)	0.169
Lipid profile			
Triglycerides, mmol/L	1.08 (0.77–1.47)	1.22 (0.87–1.66)	0.148
Total cholesterol, mmol/L	3.08 (2.60–3.81)	3.17 (2.65–3.75)	0.757
LDL cholesterol, mmol/L	1.90 (1.38–2.46)	1.81 (1.36–2.26)	0.239
HDL cholesterol, mmol/L	0.88 (0.66–1.25)	0.84 (0.59–1.10)	0.073
Composite scores			
Monocyte-to-albumin ratio	0.02 (0.01–0.02)	0.02 (0.01–0.02)	0.263
Neutrophil-to-albumin ratio	0.22 (0.14–0.34)	0.25 (0.17–0.37)	0.163

Data are presented as median (interquartile range) or n (%). P values were derived from the Wilcoxon rank-sum test for continuous variables and the Pearson chi-square test for categorical variables. Abbreviations: ALT, alanine aminotransferase; AST, aspartate aminotransferase; BNP, B-type natriuretic peptide; CK, creatine kinase; HDL, high-density lipoprotein; hs-CRP, high-sensitivity C-reactive protein; LDH, lactate dehydrogenase; LDL, low-density lipoprotein; $PaCO_2$, partial pressure of arterial carbon dioxide; PaO_2, partial pressure of arterial oxygen; RDW, red blood cell distribution width; SpO_2, peripheral oxygen saturation.

Supplementary Table S3. Variance inflation factors for predictors included in the final multivariable logistic regression model.

Variable	VIF
Age	1.05138246823816
BMI	1.03324502585397
Barthel_Score	1.07161006860756
SOFA_Score	1.04865909637844
Sodium	1.03637698425617
Albumin	1.03188384016769
HALP_Score	1.02930370221232

VIF values >5 were considered indicative of multicollinearity. All VIF values were close to 1, indicating no evidence of multicollinearity among predictors in the final model.

Abbreviations: VIF, variance inflation factor; BMI, body mass index; SOFA, Sequential Organ Failure Assessment; HALP, hemoglobin–albumin–lymphocyte–platelet.

Supplementary Table S4. Internal validation of model performance using bootstrap resampling (1,000 iterations).

Metric	Original cohort	Bootstrap mean (95% CI)
AUC	0.803	0.797 (0.785–0.804)
Optimism	—	0.018
Optimism-corrected AUC	—	0.785
Accuracy	0.725	0.712 (0.681–0.740)

Bootstrap resampling was performed with 1,000 iterations. Abbreviations: AUC, area under the receiver operating characteristic curve; CI, confidence interval.

Supplementary Table S5. Sensitivity analysis using proxy GLIM criteria.

**Metric**	**Value**
GLIM-positive patients, n (%)	126 (39.0%)
AUC	0.708
95% CI	0.650–0.766
Accuracy	0.641
Sensitivity	0.627
Specificity	0.650
PPV	0.534
NPV	0.731

Proxy GLIM positivity was defined as the presence of low BMI as the phenotypic criterion and sepsis as the etiologic criterion. Low BMI was defined as BMI <18.5 kg/m^2 for patients aged <70 years and BMI <20.0 kg/m^2 for those aged ≥70 years. Because direct measures of muscle mass or muscle function were unavailable, this analysis should be interpreted as exploratory rather than as a definitive GLIM-based diagnosis of malnutrition.

Abbreviations: AUC, area under the receiver operating characteristic curve; BMI, body mass index; CI, confidence interval; GLIM, Global Leadership Initiative on Malnutrition; NPV, negative predictive value; PPV, positive predictive value.

Supplementary Table S6. Discriminative performance of the model across age-stratified subgroups.

Age cutoff	Subgroup	n	AUC (95% CI)
70 years	<70 years	70	0.833 (0.730–0.937)
	≥70 years	253	0.783 (0.726–0.840)
80 years	<80 years	176	0.834 (0.773–0.896)
	≥80 years	147	0.760 (0.681–0.839)
90 years	<90 years	286	0.806 (0.754–0.857)
	≥90 years	37	0.771 (0.616–0.927)

AUC remained above 0.75 across all age-stratified subgroups. The highest discriminative performance was observed in the <80 years subgroup (AUC = 0.834). Abbreviations: AUC, area under the receiver operating characteristic curve; CI, confidence interval.

Supplementary Table S7. Direct comparison of discriminative performance between the nomogram and the mNUTRIC score.

**Model**	**AUC**	**95% CI**	***P* value***
Nomogram	0.803	0.755–0.852	Reference
mNUTRIC Score	0.658	0.595–0.722	< 0.001

*P value was calculated using DeLong's test for correlated receiver operating characteristic curves, comparing the AUC of the mNUTRIC score with that of the nomogram.

Abbreviations:

AUC, area under the receiver operating characteristic curve; CI, confidence interval; mNUTRIC, modified Nutrition Risk in the Critically Ill.

Supplementary Table S8. Sensitivity analysis with additional adjustment for inflammatory markers.

**Variable**	**Original Model**	**+ hs-CRP**	**+ hs-CRP + PCT**
	**OR (95% CI)**	**OR (95% CI)**	**OR (95% CI)**
Age, years	1.03 (1.00–1.06)	1.03 (1.00–1.06)	1.03 (1.00–1.06)
BMI, kg/m^2	0.88 (0.78–0.98)	0.88 (0.78–0.98)	0.88 (0.78–0.98)
Barthel Index	0.97 (0.96–0.98)	0.97 (0.96–0.98)	0.97 (0.96–0.98)
SOFA score	1.25 (1.03–1.53)	1.25 (1.03–1.53)	1.25 (1.03–1.53)
Sodium, mmol/L	0.96 (0.91–1.00)	0.95 (0.91–1.00)	0.95 (0.91–1.00)
Albumin, g/L	0.94 (0.89–0.99)	0.93 (0.89–0.99)	0.93 (0.89–0.99)
HALP score	0.98 (0.96–1.00)	0.98 (0.96–1.00)	0.98 (0.96–1.00)
**Model AUC**	**0.803**	—	**0.805**

Values are presented as odds ratios with 95% confidence intervals unless otherwise indicated. hs-CRP and PCT were log-transformed before inclusion in the sensitivity models. The similarity of effect estimates across models suggests that the associations between the seven predictors and NRS-2002-defined nutritional risk were not materially explained by measured acute inflammatory markers. Abbreviations: AUC, area under the receiver operating characteristic curve; BMI, body mass index; CI, confidence interval; HALP, hemoglobin–albumin–lymphocyte–platelet; hs-CRP, high-sensitivity C-reactive protein; OR, odds ratio; PCT, procalcitonin; SOFA, Sequential Organ Failure Assessment.

Supplementary Table S9. Performance of the nomogram in the hold-out test set from split-sample validation.

**Metric**	**Value**
Test set sample size, n	96
AUC	0.779
95% CI	0.683–0.875
Accuracy	0.708
Sensitivity	0.742
Specificity	0.692
PPV	0.535
NPV	0.849

The cohort was randomly split into a training set and a hold-out test set at a 70:30 ratio. Model performance was evaluated in the hold-out test set. Sensitivity, specificity, accuracy, PPV, and NPV were calculated using the classification threshold determined in the training set.

Abbreviations: AUC, area under the receiver operating characteristic curve; CI, confidence interval; NPV, negative predictive value; PPV, positive predictive value.

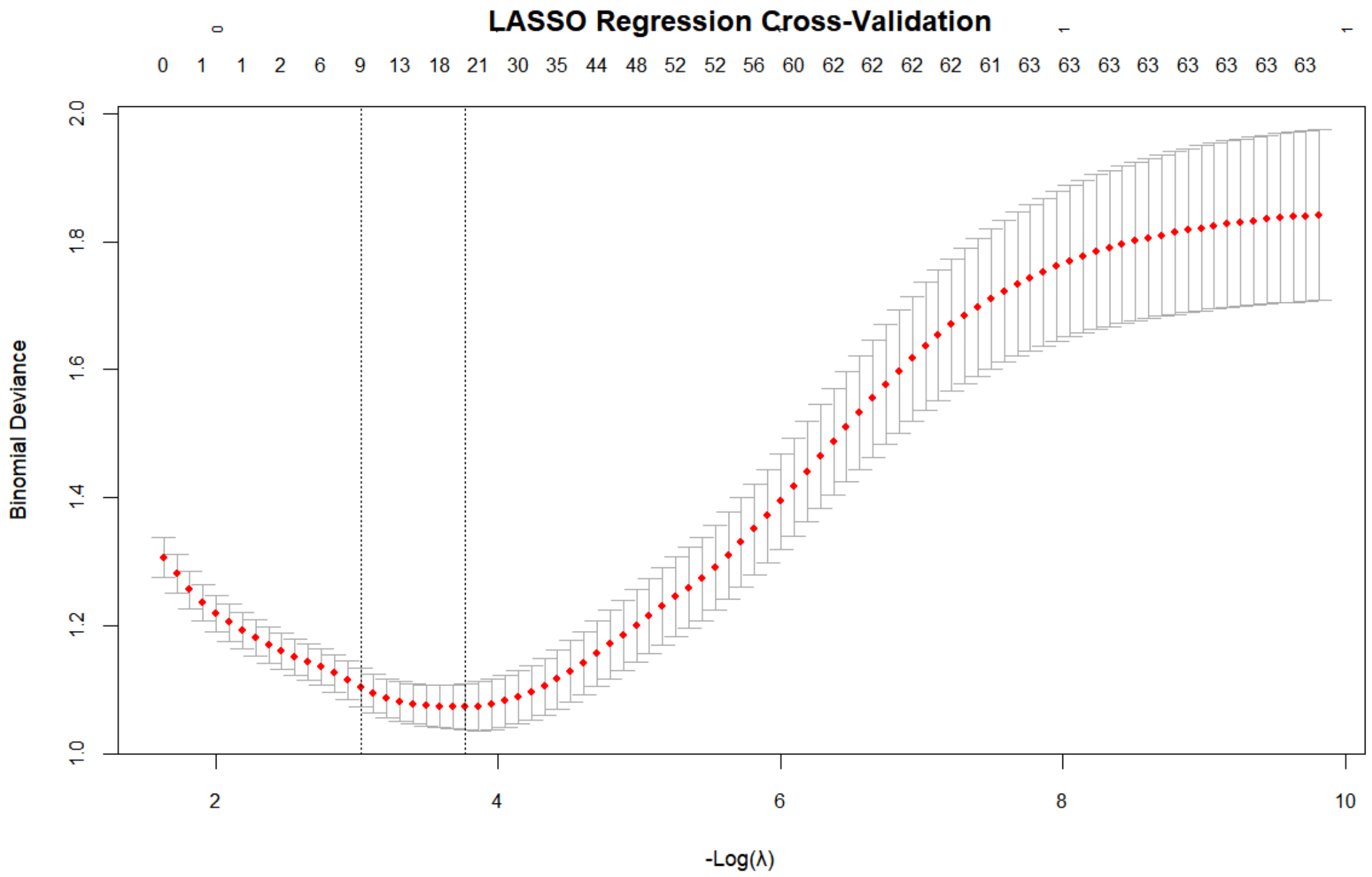

Supplementary Figure 1. Selection of the penalty parameter in LASSO regression. Ten-fold cross-validation plot showing the relationship between the penalty parameter (λ) and binomial deviance. The left and right vertical dashed lines indicate the values of λ that minimize the cross-validated error (λ_{min}) and the largest value within one standard error of the minimum (λ_{1se}), respectively. The λ_{1se} criterion was selected to obtain a more parsimonious model.

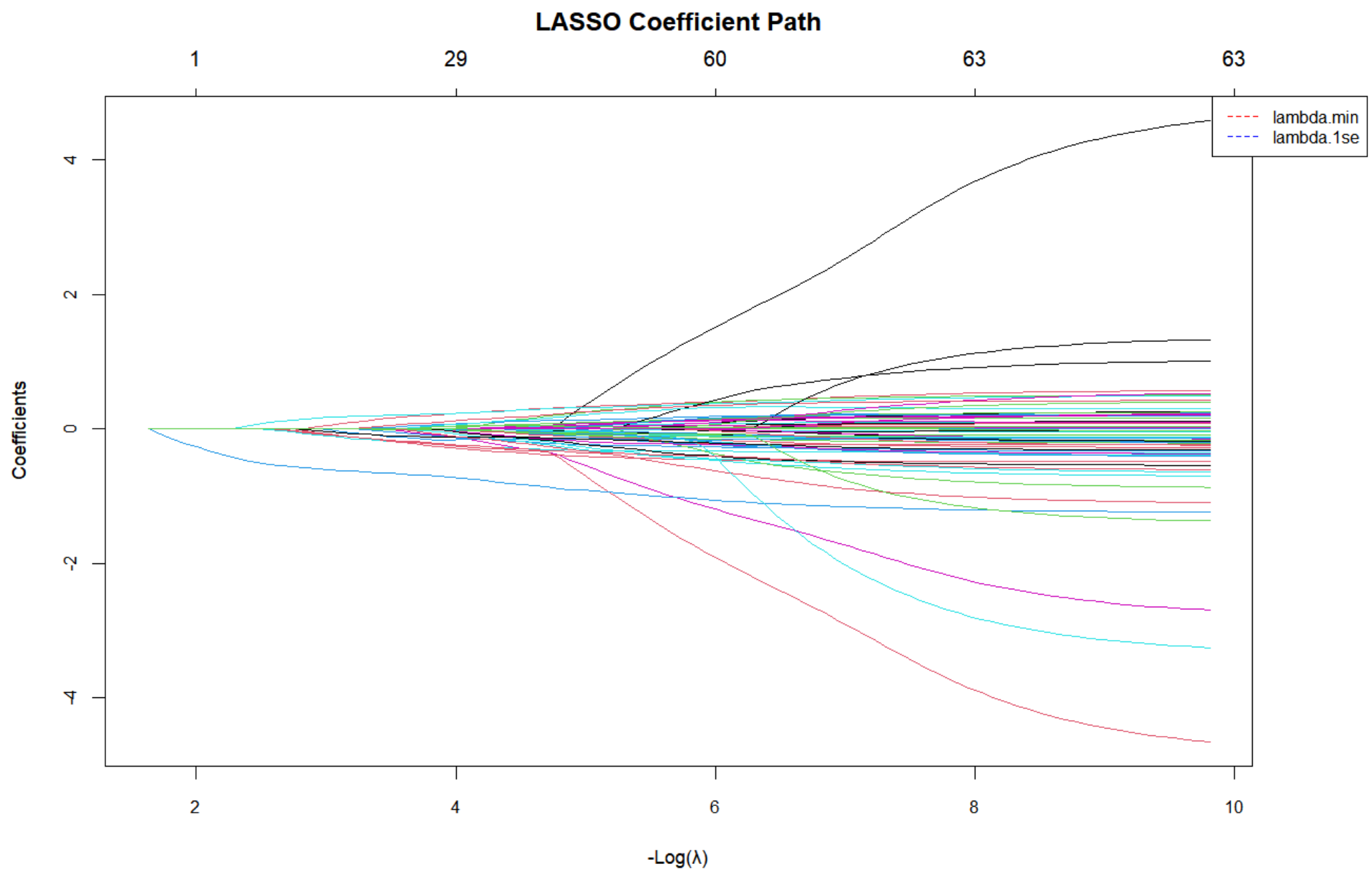

Supplementary Figure 2. Coefficient profiles in LASSO regression. LASSO coefficient path plot showing the trajectories of regression coefficients for candidate variables as a function of the penalty parameter (λ), plotted against $-\log(\lambda)$. Each line represents one variable. As λ increases, coefficients are progressively shrunk towards zero. The vertical dashed lines indicate the values of λ corresponding to λ_{min} and λ_{1se}. Variables with non-zero coefficients at λ_{1se} were retained for subsequent multivariable modelling.

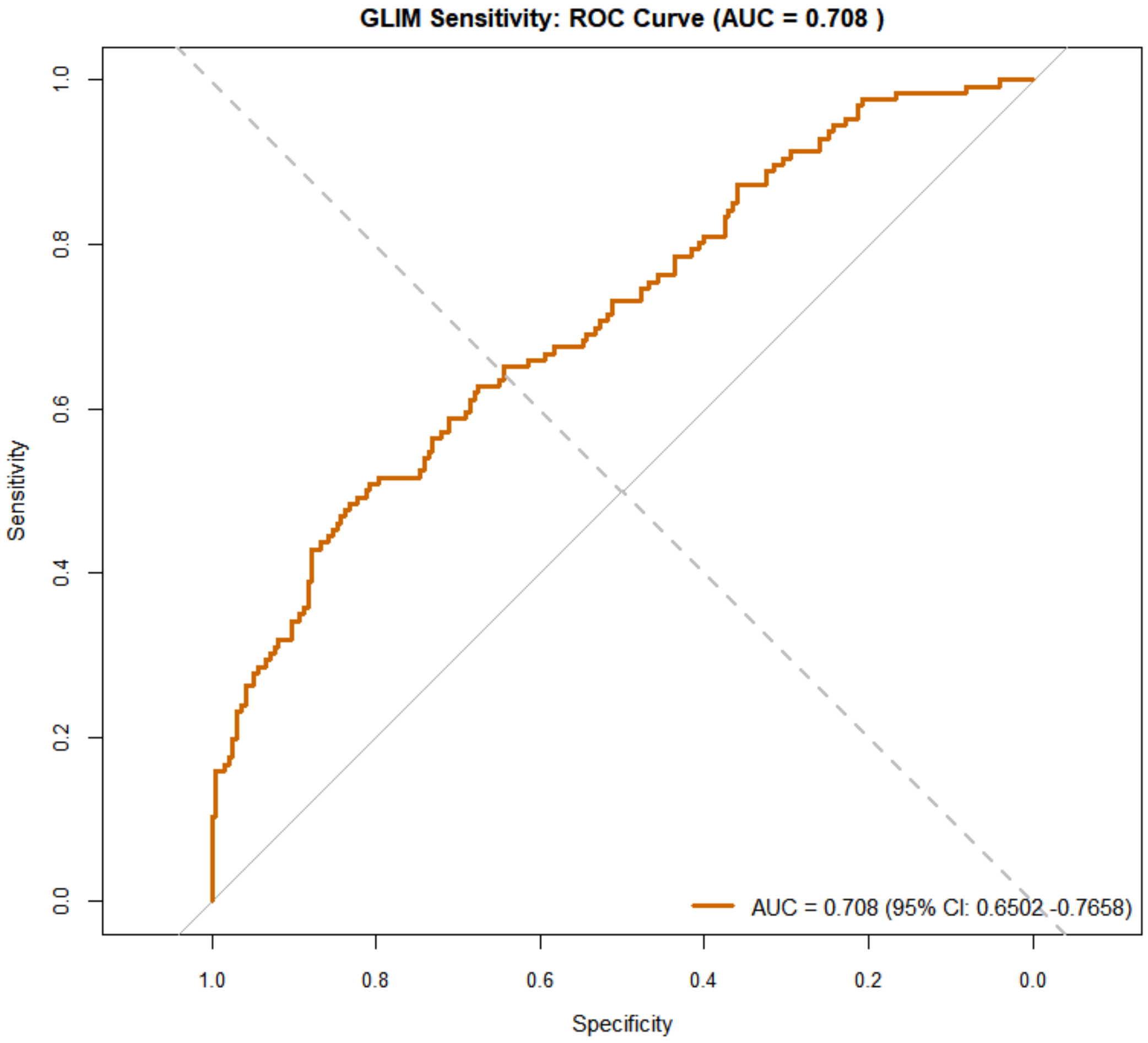

Supplementary Figure S3. Receiver operating characteristic curve for the proxy GLIM sensitivity analysis. The nomogram yielded an AUC of 0.708 (95% CI, 0.650–0.766) for identifying proxy GLIM-positive patients, defined using low BMI as the phenotypic criterion and sepsis as the etiologic criterion. This result indicates moderate discriminative performance against a BMI-based proxy GLIM outcome and should be interpreted as exploratory because direct measures of muscle mass or muscle function were unavailable.

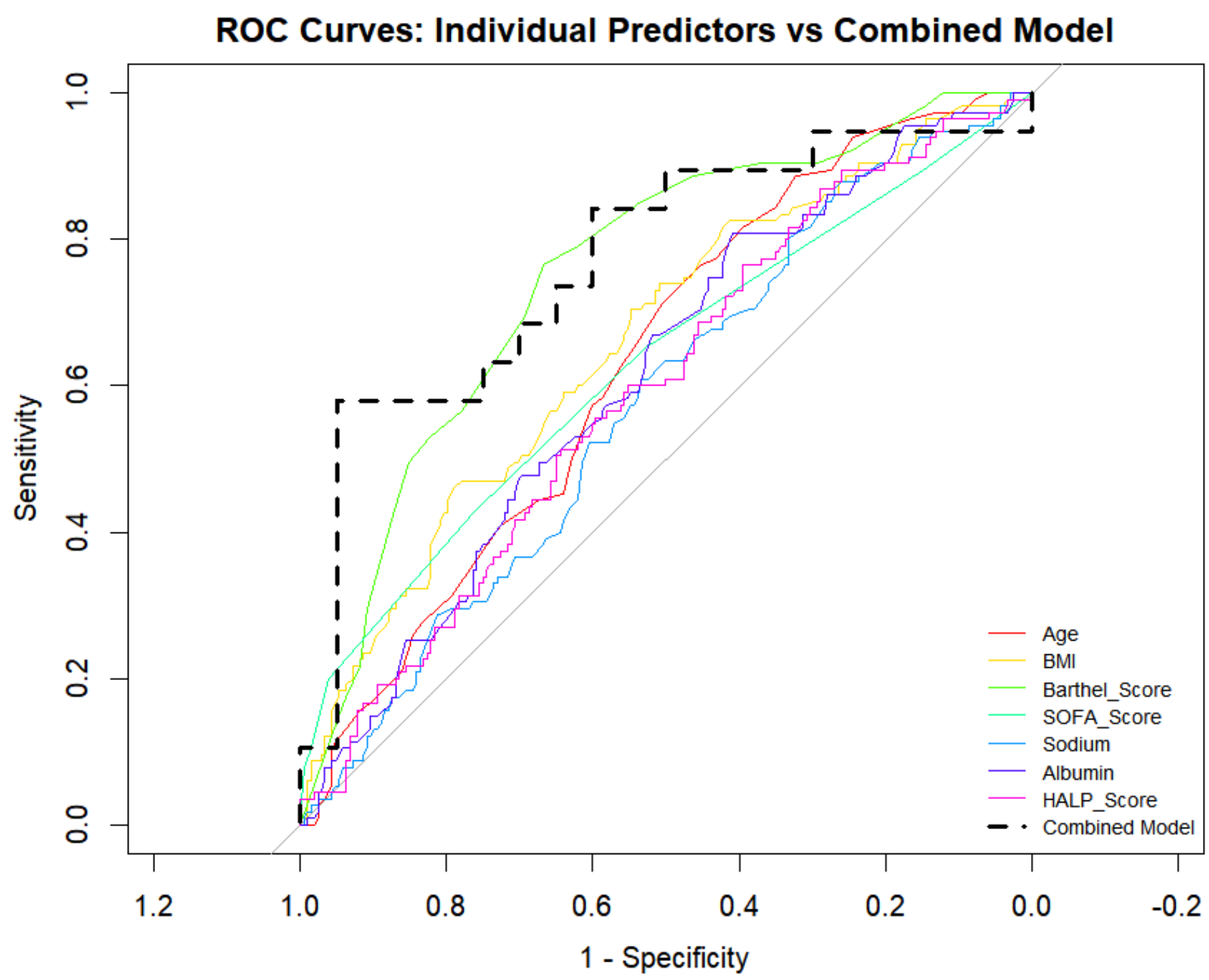

Supplementary Figure S4. Comparison of discriminative performance between individual predictors and the combined model. Receiver operating characteristic (ROC) curves comparing the discriminative ability of individual predictors with that of the combined multivariable model for predicting nutritional risk. The combined model showed improved discrimination relative to individual predictors.

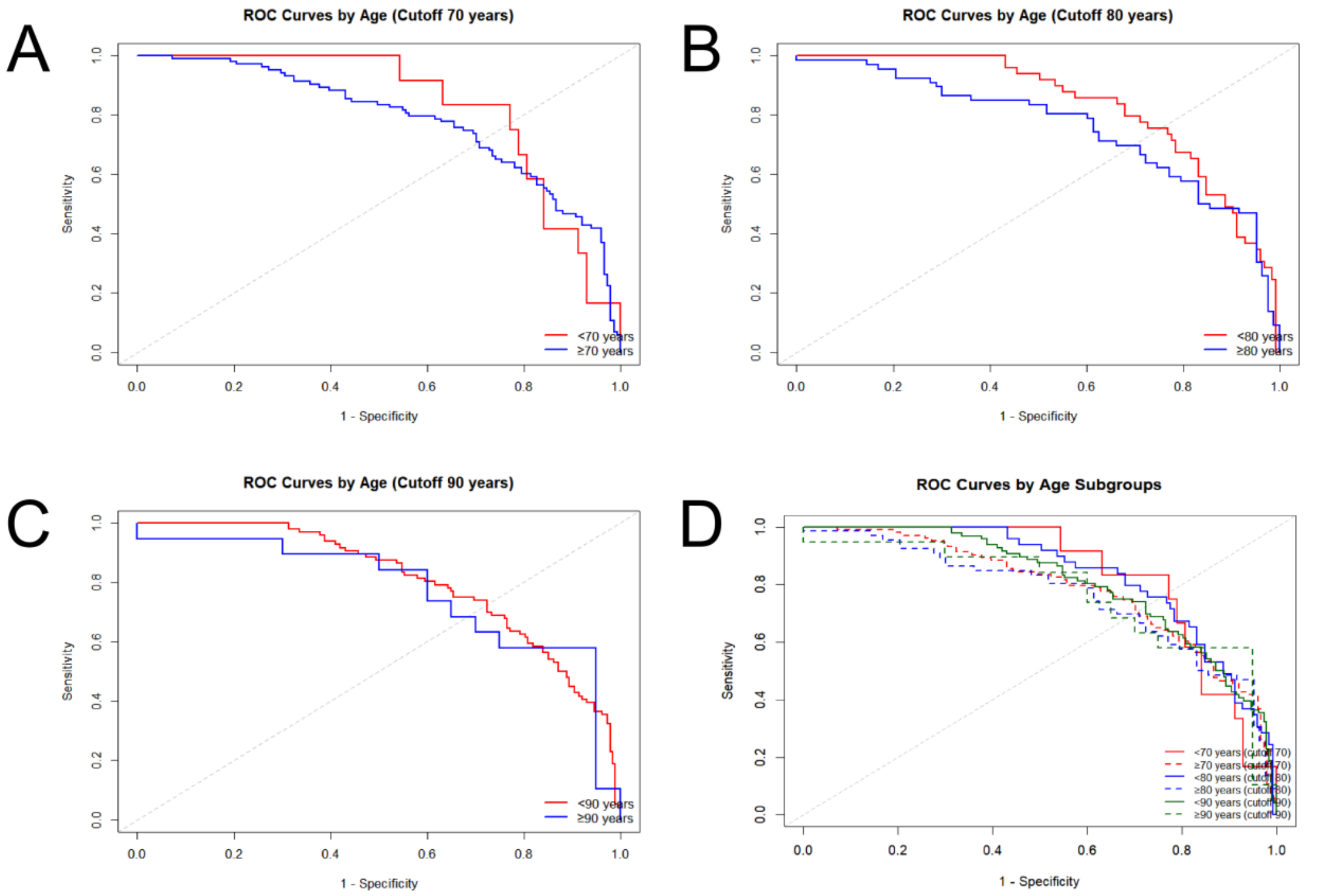

Supplementary Figure S5. Discriminative performance of the model across age-stratified subgroups. Receiver operating characteristic (ROC) curves of the multivariable model for predicting nutritional risk across age-based subgroups: (A) <70 vs. ≥70 years; (B) <80 vs. ≥80 years; (C) <90 vs. ≥90 years; and (D) combined comparison across all thresholds. The model showed broadly consistent discrimination across age strata.

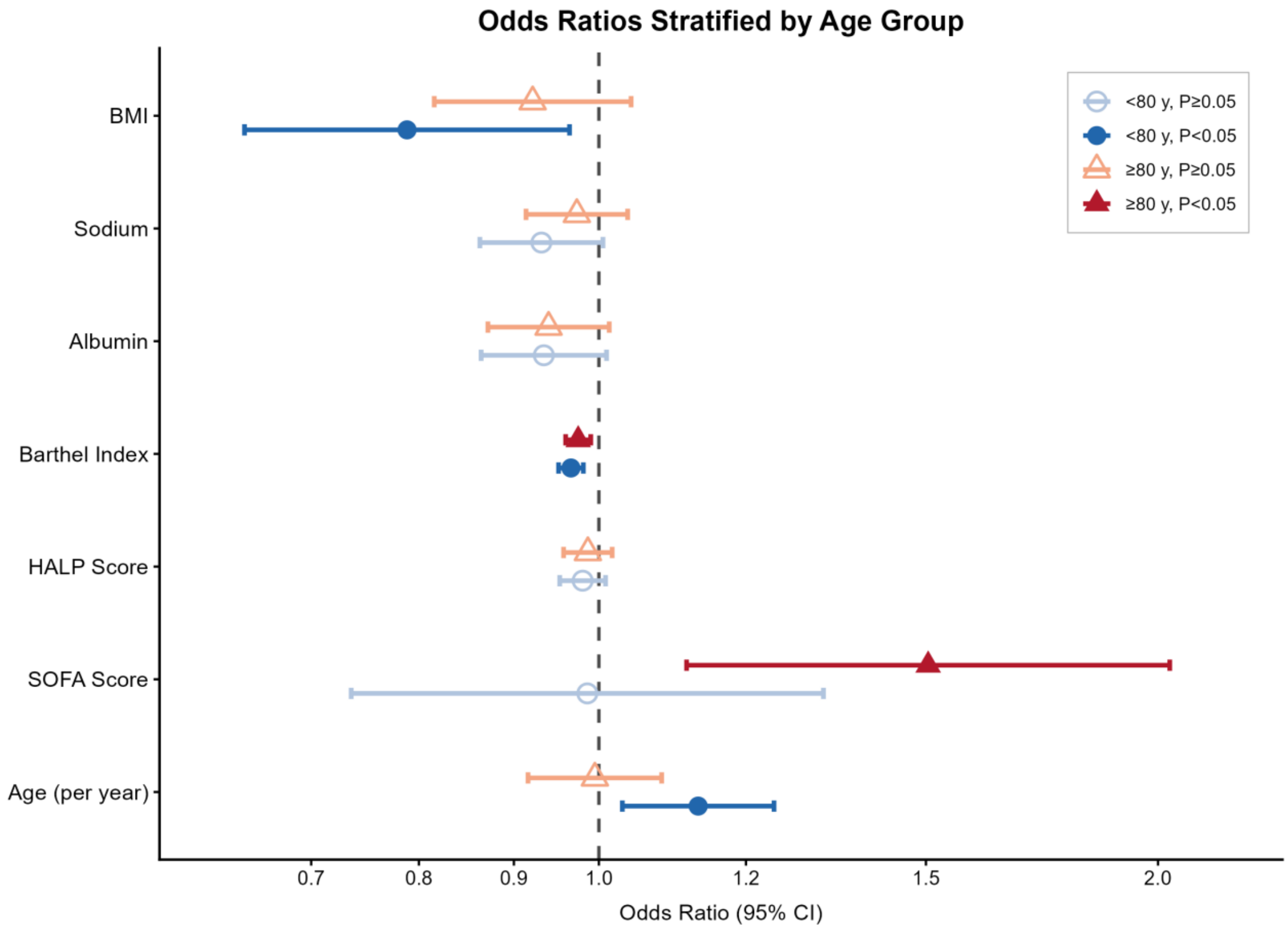

Supplementary Figure S6. Age-stratified analysis of predictors for malnutrition (<80 vs. ≥80 years). Forest plot showing adjusted odds ratios (ORs) and 95% confidence intervals from multivariable logistic regression models fitted separately in patients aged <80 years (n = 174) and ≥80 years (n = 149), including the same seven predictors as in the final combined model (age, BMI, Barthel Index, SOFA score, sodium, albumin, and HALP score). A significant interaction between age and age group was observed (P for interaction = 0.011, Type II Wald χ^2 test). Age was associated with malnutrition in patients aged <80 years (OR per year = 1.13, 95% CI 1.03–1.24, P = 0.010), but not in those aged ≥80 years (OR = 0.995, 95% CI 0.92–1.08, P = 0.906). All other predictors showed consistent effect directions across age strata (all P for interaction > 0.05). Abbreviations: BMI, body mass index; SOFA, Sequential Organ Failure Assessment; HALP, hemoglobin, albumin, lymphocyte, and platelet score; CI, confidence interval; OR, odds ratio.

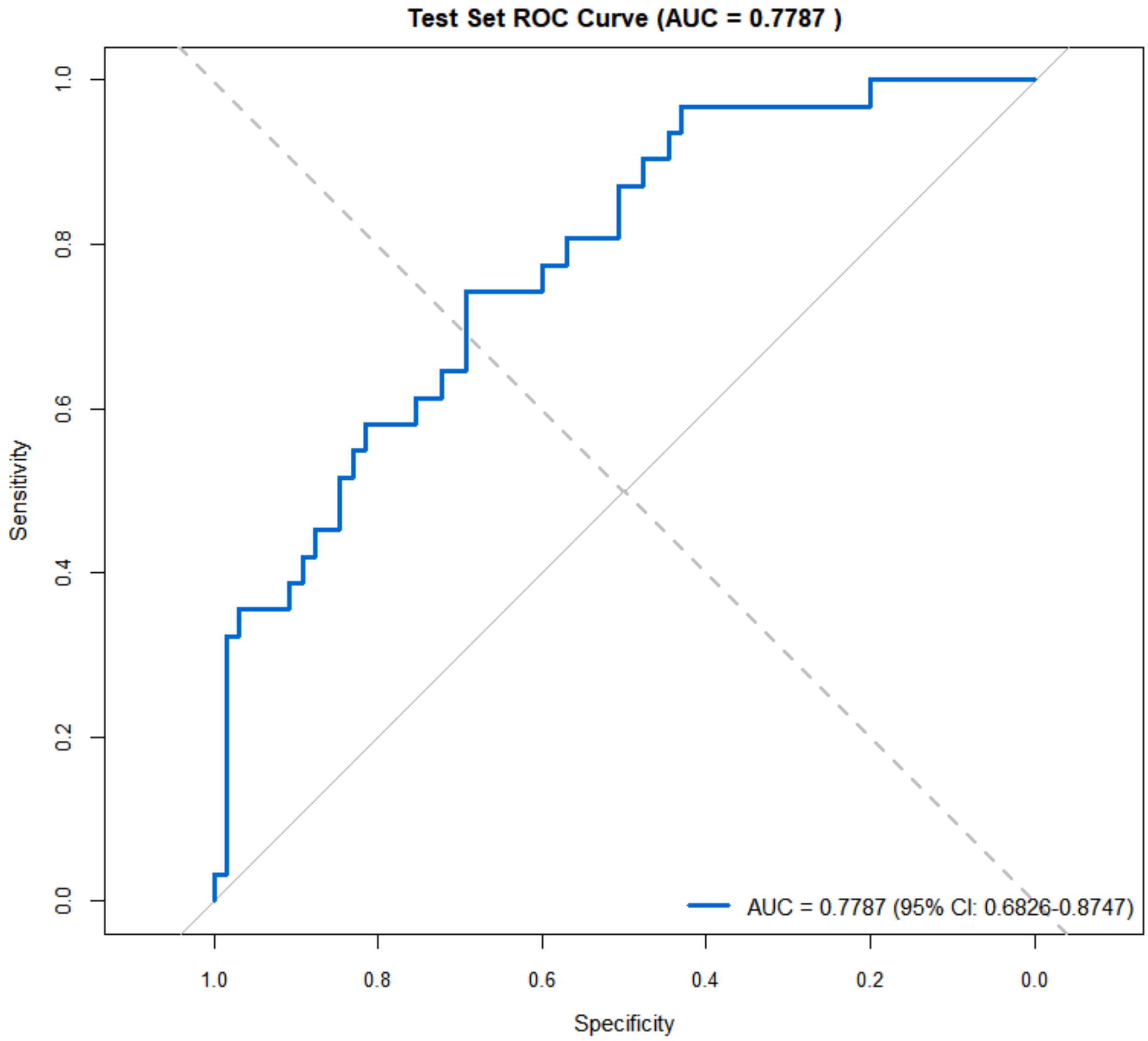

Supplementary Figure S7. Receiver operating characteristic curve of the nomogram in the hold-out test set. In split-sample internal validation, the cohort was randomly divided into a training set and a hold-out test set at a 70:30 ratio. The nomogram achieved an AUC of 0.779 (95% CI, 0.683–0.875) in the hold-out test set (n = 96), indicating acceptable discrimination.

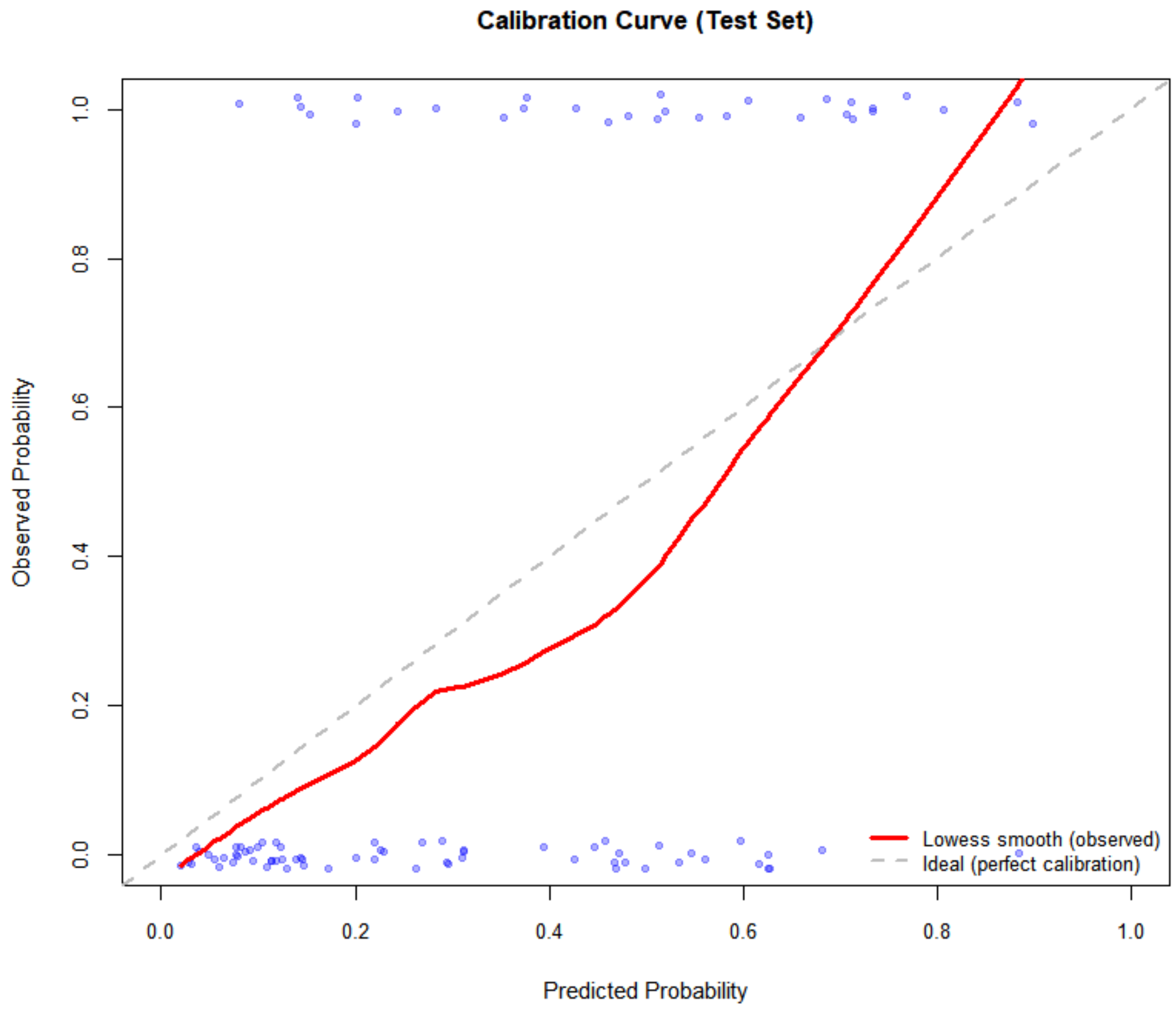

Supplementary Figure S8. Calibration curve of the nomogram in the hold-out test set. The calibration curve compares predicted and observed probabilities of NRS-2002-defined nutritional risk in the hold-out test set from split-sample internal validation. The LOWESS-smoothed curve showed overall agreement with the ideal reference line but with visible deviations across parts of the predicted-risk range, indicating acceptable but imperfect calibration.
